# Effect of IMOD^™^ on the inflammatory process after acute ischemic stroke: a randomized clinical trial

**DOI:** 10.1186/2008-2231-21-26

**Published:** 2013-03-20

**Authors:** Mehdi Farhoudi, Mahdi Najafi-Nesheli, Mazyar Hashemilar, Ata Mahmoodpoor, Ehsan Sharifipour, Behzad Baradaran, Aliakbar Taheraghdam, Daryoush Savadi-Oskouei, Homayoun Sadeghi-Bazargani, Elyar Sadeghi-hokmabadi, Hosein Akbari, Reza Rikhtegar

**Affiliations:** 1 Neuroscience Research Center (NSRC), Imam Reza Hospital, Tabriz University of Medical Sciences, Tabriz, Iran; 2 Department of Anesthesiology and critical care medicine, Imam Reza Hospital, Tabriz University of Medical Sciences, Tabriz, Iran; 3 Student Research Committee, Tabriz University of Medical Sciences, Tabriz, Iran

**Keywords:** Ischemic cerebro-vascular accident, IMOD™, Inflammatory markers

## Abstract

**Background and purpose of the study:**

Considering the role of inflammation in acute cerebrovascular accidents, anti-inflammatory treatment has been considered as an option in cerebrovascular diseases. Regarding the properties of Setarud (IMOD™) in immune regulation, the aim of the present study was to evaluate the role of this medication in treating patients with acute ischemic stroke.

**Methods:**

In this randomized clinical trial, 99 patients with their first ever acute ischemic stroke were divided into two groups of IMOD™ (n = 49) and control (n = 50). The control group underwent routine treatment and the intervention group underwent routine treatment plus daily intermittent infusion of IMOD™ (250mg on the first day and then 375mg into DW5% serum during a 30-minute period for 7 days). The serum levels of inflammatory markers were evaluated on the first day (baseline) and on 4th and 7th days. Data were analyzed and the results were compared.

**Results and major conclusion:**

58 males (58.6%) and 41 females (41.4%) with a mean age of 67.00 ± 8.82 years, who had their first ever stroke attack, were enrolled in this trial. Treatment with IMOD™ showed a decreasing trend in IL-6 levels compared to the control group (p = 0.04). In addition, the treatment resulted in the control of increasing serum levels of hsCRP after 7 days compared to the control group (p = 0.02). There was an insignificant decrease in TNF-α and IL-1 levels in the IMOD™ group. Considering the prominent role of inflammation after an ischemic cerebral damage, it appears that treatment with IMOD™ improves the inflammatory profile. Therefore, IMOD™ (Setarud) might be considered as a therapeutic option in the acute ischemic stroke. However, future studies are necessary on its long-term results and clinical efficacy.

## Background

Cerebrovascular accident is the main etiologic factor for disability in adults and the second most important cause of death worldwide [1]. Based on available evidence, a strong inflammatory reaction is induced subsequent to acute CVA, which has a great role in cerebral injury, demonstrating a significant interaction between the immune and nervous systems [2,3].

This inflammatory reaction is mediated by various cells, molecules and cytokines [3]; cytokines are upregulated in the brain after the stroke and are expressed not only in the immunologic cells but also in the glial cells and neurons [4].

The most extensively studied cytokines associated with stroke are IL-1β, IL-10, IL-6 and TNF-α. IL-1β and TNF-α, proinflammatory cytokines secreted by the activated immune cells in the ischemic area, induce the inflammatory process and facilitate the inflammatory cascade by inducing the expression of inflammatory molecules. These molecules recruit more leukocytes to the affected ischemic area, give rise to the loss of more nerve cells, cause cerebral tissue and expand cerebral infarction [5,6].

Considering the role of cytokines in neurologic inflammation, these inflammatory mediators can be the target of neuroimmunomodulatory treatment [7]. Immunomodulatory medication is a substance that alters the ability of the immune system to produce antibodies or sensitized cells that recognize and react with the antigens that have initiated their production.

Setarud (IMOD™) is a combination of the extracts of Tanacetum vulgare, Rosa canina and Urtica dioica plant species, which has been enriched with selenium. The plant content of this medication has anti-inflammatory and immunoregulatory properties and selenium has a protective effect against oxidative stress. Extracts from Urtica dioica may prevent maturation of myeloid dendritic cells and reduce T cell responses. Multiple in vitro and in vivo studies in animal models and also in human has shown that it decreases TNF-α, IFN-γ and IL-2 levels and its effect in some clinical situations, such as experimental inflammatory bowel diseases, immunogenic type-1 diabetes in mouse and also in patients with sepsis and in HIV patients, has been evaluated due to its immunoregulatory properties [8-15]. Shirazi and colleagues in an in vitro study concluded that the dose-dependent inhibitory effect of Setarud on TLR stimulated B lymphocytes implies its potential therapeutic implication in B lymphocyte mediated autoimmune diseases and B-cell malignancies [16]. In addition, the herbal content of this medication can exhibit anti-inflammatory, anti-viral and immonomodulatory effects [17-20].

Considering the inflammatory changes during the acute phase of ischemic stroke and its central role on disease outcome, anti-inflammatory treatment might be an appropriate option in such patients; in addition, considering the role of IMOD™ in immunoregulation, the aim of the present study was to evaluate the effect of this medication on chief inflammatory biomarkers in patients with acute ischemic cerebrovascular accidents.

## Methods

After approval of the ethics committee of Tabriz University of Medical Sciences, 99 patients with first attack of acute ischemic stroke (AIS), hospitalized in Tabriz Imam Reza and Razi Hospitals from September 2011 to March 2012, were enrolled in the study (using convenient sampling), and were randomly divided into two groups, with an allocation sequence based on a block size of fifty, generated with computer random-number generator. Allocation was concealed by use of sequentially numbered black envelopes. (IRCT No: IRCT 201108202195N2)

The AIS was initially diagnosed using Cincinnati Prehospital Stroke Scale (Facial droop, Arm drift and Speech) and confirmed based on clinical criteria and imaging techniques as used in TOAST Subtype Classification System [21]. The exclusion criteria were: age over 80 or under 18; previous history of stroke; a previous neurological deficit of any cause; cancer or any other severe disease; a rapid improvement in signs and symptoms before the institution of treatment; a baseline NIHSS (National Institutes of Health Stroke Scale) below5; elapse of less than 3 hours and more than 24 hours after the appearance of signs and symptoms; refusal to sign an informed consent form and a history of taking immunomodulatory medications such as immunoglobulin’s or steroids.

All the patients who were available during the specified time interval and had the inclusion criteria were enrolled in the study. Since it was not possible to use placebo because of technical and ethical limitations, blinding was carried out through masked evaluation and any unmasked condition was taken into account during statistical analysis.

In the present study the subjects were divided into two randomized groups of case and control using stratified block randomization technique. The subjects in the control groups underwent a routine treatment protocol (according to the protocol of the University Department of Neurology); the subjects in the case group underwent routine treatment protocol plus daily intermittent infusion of IMOD™ (250mg on the first day and 375mg on the subsequent days into DW5% serum) during 30 minutes for 7 days. Previous studies showed that IMOD didn’t have any side effect and only one case of phlebitis was reported [9]. So we used DW5% for its dilution and a duration of 30 minutes time for IMOD injection to reduce the probability of phlebitis. Both groups equally received routine therapeutic, supportive and rehabilitative measures. All study participants had blood samples taken at the first, fourth and seventh day of admission. Non-fasting blood was collected and within 30 minutes, the blood was centrifuged for 15 minutes at 3000 rotations per minute at room temperature. Subsequently, U-CyTech kit was used for determination of serum inflammatory markers by ELISA method. The levels of TNF-α, IL-6 and IL-1, and also platelets, WBC and High-sensitive CRP were determined on the first day of disease presentation (before treatment) and on the 4th and 7th days, by laboratory procedures in the hospital immunology department.

Informed consent was obtained from all the subjects. All the subjects were thoroughly under supervision in relation to the occurrence of any complication and all the equipment necessary for the control of acute conditions were available in the department.

### Statistical analysis

STATA 11® statistical software was used to provide a randomization list in two equal 50-member blocks. Data were analyzed using SPSS 16® statistical software. Independent t-test, or as required Mann–Whitney U test, was used for quantitative variables. Repeated measures ANOVA were used to evaluate changes in quantitative variables throughout the study. Statistical significance was defined at *P* < 0.05.

## Results

In the present study, 99 patients with their first ever stroke consisting of 58 males (58.6%) and 41 females (41.4%) with a mean age of 67.00 ± 8.82 years, were enrolled. Forty-nine patients (49.5%) were randomly placed in the IMOD™ group and 50 patients (50.5%) were placed in the control group. Tables 1 and 2 presents the baseline data of the two groups. As it is shown the two groups were matched in relation to all the baseline findings.

**Table 1:** Baseline findings in the two groups under study

	**IMOD™ group**	**Control group**	***P *value**
Age	67.00 ± 8.16	67.00 ± 9.50	0.90
Gender (male)	30 (61.2%)	28 (56.0%)	0.68
IHD	15(30.6%)	13 (26.0%)	0.66
HTN	38(77.6%)	35 (70.0%)	0.49
DM	6(12.2%)	10(20.0%)	0.41
CHF	1(2.0%)	2(4.0%)	0.90
Smoking	9(18.4%)	15(30.0%)	0.24
Atrial Fibrillation	3(6.1%)	4(8.0%)	0.9
Hyperlipidemia	7(14.3%)	13(26.0%)	0.21
ASA use	18(36.7%)	20(40.0%)	0.77
ACEI/ARB use	28(57.1%)	26(52.0%)	0.72
Statin use	7(14.3%)	13(26.0%)	0.21

DM: Diabetes Mellitus, CHF: Congestive Heart Failure, IHD: Ischemic Heart Failure, HTN: Hypertension.

ACEI/ARB: Angiotensin Converting Enzyme Inhibitors/Angiotensin Receptor Blockers.

Data are mean ± SE.

**Table 2:** Baseline clinical characteristics based on NIHSS in the two groups under study

	**IMOD™ group**	**Control group**	***P *value**
NIHSS	10.59 ± 5.56	11.04 ± 6.56	0.71
Facial weakness	1.57 ± 0.54	1.50 ± 0.54	0.51
Arm weakness	2.33 ± 1.12	2.30 ± 1.24	0.91
Leg weakness	2.02 ± 1.14	1.96 ± 1.27	0.80
Speech difficulty	1.33 ± 0.47	1.36 ± 0.56	0.75

NIHSS: National Institute of Health Stroke Scale.

Data are mean ± SE.

The mean duration of time between the appearance of signs and symptoms and the institution of treatment was 12.92 ± 3.80 hours in the IMOD™ group, with a median of 13 hours. The shortest and longest times were 5 and 21 hours, respectively. The patients in the IMOD™ group were aware, lethargic, obtunded and stuporous in 85.7%, 8.20%, 2% and 4.10% of cases, respectively at the time of referral; the percentages above were 70%, 16%, 4% and 6%, respectively, in the control group, with 4% of comatose patients in this group. In relation to stroke types in the TOAST classification [21], in the IMOD™ group the vessel involvements were as follows: 26 cases (53.1%) of large vessels, 5 cases (10.2%) of cardioembolic and 18 cases (36.7%) of lacunar; in the control group the percentages above were as follows, respectively: 26 cases (52%), 6 cases (12%) and 18 cases (36%). No significant differences were observed between the two groups (p = 0.93).No major adverse effect of IMOD injection was seen except for three cases of superficial phlebitis that resolved by conservative measures.

Considering the differences in the laboratory findings between the two groups (insignificant at baseline), the effects of the medications were evaluated by assessing the changes observed.

**Figure 1:**
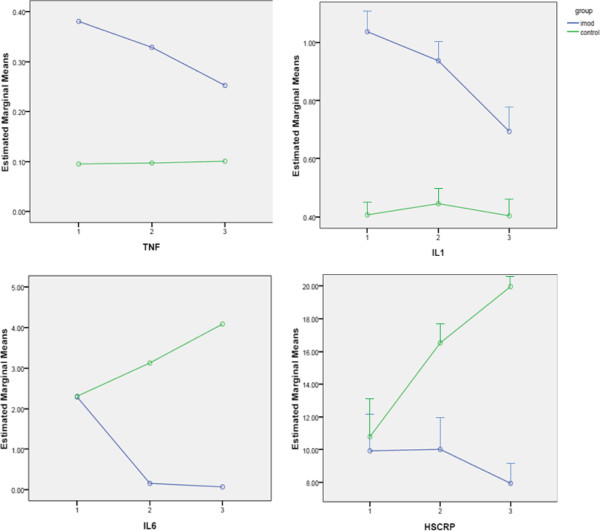
**Changes in serum levels of TNF-α, IL-1, IL-6 and hsCRP in different days of study.** Data are mean ± SE. Difference between two groups is not significant for TNF-α (P = 0.10) and IL-1 (P = 0.20) but is significant for IL-6 (P = 0.04) and hsCRP (P = 0.02).

Figure 1 shows changes in IL-1 levels in the two groups during the study. As the figure shows IL-1 exhibited a decreasing trend in the IMOD™ group; however, it exhibited almost constant variations in the control group. No significant differences were observed between the two groups (p = 0.20).

As shown in Figure 1, IL-6 levels increased significantly in the control group until the 7th day. However, treatment successfully decreased the levels of this marker (p = 0.04).

Figure 1 also shows changes in TNF-α level in the two groups; TNF-α levels exhibited a mild increase in the control group by the 7th day but decreased in the IMOD™ group; however, treatment did not significantly decrease TNF-α levels (p = 0.10).

**Figure 2:**
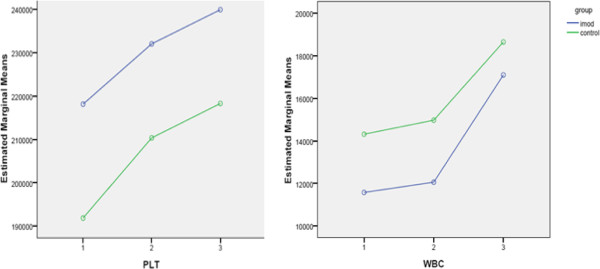
**Changes in counts of Platelet (PLT) and White Blood Cells (WBC) in different days of study.** Data are mean ± SE. Difference between two groups is not significant for PLT (P = 0.13) and WBC (P = 0.71).

Figure 2 shows variations in hsCRP levels in the two groups of study; hsCRP levels exhibited an almost constant level after Setarud was administered and then decreased. However, in the control group an increasing trend was observed before the 7th day. Treatment successfully controlled increasing hsCRP levels (p = 0.02).

As shown in Figure 2, WBC and platelet counts increased in both groups throughout the 7 days period, indicating that treatment did not influence these two markers (p = 0.71 and p = 0.13, respectively).

Patients in each group were divided into two groups of NIHSS ≥ 15 and NIHSS < 15 based on NIHSS scores. Laboratory findings in the IMOD™ group showed that only hsCRP levels were significantly higher in patients with NIHSS ≥ 15 at the time of referral (9.07 ± 1.75 vs. 21.92 ± 5.38) and 4 days after the treatment was instituted (10.95 ± 1.73 vs. 22.00 ± 4.69) (p = 0.01 and p = 0.02, respectively). Evaluation of laboratory findings in the control group also revealed that only hsCRP levels were significantly higher at the time of referral (9.84 ± 2.03 vs. 18.54 ± 96) and 4 days after the institution of treatment (27.57 ± 3.98 vs. 16.05 ± 2.38) in patients with NIHSS ≥ 15 (p = 0.04 and p = 0.04, respectively).

Considering the difference in biomarker levels during treatment in each group, just the decrease of first day’s serum IL6 level in comparison to 4^th^ and 7^th^ days’ serum IL6 level in treatment group was significant (p = 0.023 and p = 0.049, respectively) and also treatment successfully controlled increasing hsCRP level.

## Discussion

Inflammation is a pathological hallmark of ischemic stroke and impairs outcome in patients via mechanisms which are poorly understood [22]. Central nervous system and the immune system interact in complex ways. Neuron destruction due to cerebral ischemia induces an immune response which is necessary to remove cell debris and initiate the regenerative process; however, this inflammatory response can exacerbate cerebral damage, resulting in secondary cerebral injuries [23,24]. Circulating inflammatory mediators can activate cerebrovascular endothelium or glial cells in the brain and impact on ischemic brain injury [22-25]. Cytokines are important inflammatory mediators and it is well established that both in vivo and in vitro ischemic challenges increase the release of a large number of cytokines which are involved in the necrosis of neuronal cells [26-29].

Among the large number of cytokines, TNF-α, IL-1 and IL-6, modulate tissue injury in experimental stroke and are therefore potential targets in stroke therapy. The effect of these cytokines on infarct evolution depends on their availability in the ischemic penumbra in the early phase after stroke onset [29]. Many neuroprotective agents have been effective in experimental stroke, yet few have translated into clinical application. One reason for this may be failure to consider clinical co-morbidities/risk factors in experimental models [30].

Therefore, attempts are aimed at controlling the principle inflammatory markers in acute ischemic stroke patients. Several efforts are performed to design and undertake trials of strategies that can modulate inflammation to improve outcomes of ischemic stroke patients. Multiple studies has been conducted to evaluate the effect of different medications on inflammation in AIS.

IL-1 is an established mediator of inflammation and damage in central nervous system (CNS) diseases in experimental studies. The IL-1 family consists of three main ligands: the agonists IL-1α and IL-1β, and the endogenous antagonist, IL-1Ra [31]. Denes et al. demonstrated that inhibition of IL-1 has beneficial effects on a variety of experimental paradigms of acute brain injury and is a promising clinical target in stroke. They proposed that blockade of IL-1 could be therapeutically useful in several diseases which are risk factors for stroke, and there is already considerable pre-clinical and clinical evidence that inhibition of IL-1 by IL-1 receptor antagonist may be valuable in the management of acute stroke [32]. Pradillo et al., have shown that a naturally occurring interleukin-1 receptor antagonist (IL-1Ra) is protective against ischemic brain damage in healthy animals. However, protective effects of IL-1Ra have not been determined in comorbid animals [30]. Shin et al. reported that the extent of complications and disease severity decrease with suppression of TNF-α and IL-1β [33].

Vinychuk et al., tested IL-6 in serum of 109 patients with ischemic stroke on the 1st day and 7 days after developing the disease. The decrease in concentration of IL-6 on the 7th day was found after a complex therapy with Flogensim in the study group in comparison with the control group where a traditional therapy was used. They found considerable difference in consequences of the ischemic stroke in 21 days among patients pertaining to different groups: the number of patients of the study group, with better results, increased and number of the patients of this group with no dynamic or even worsening in neurological status decreased [34].

De Aguilar-Nascimento et al. demonstrated enteral formula containing whey protein may decrease inflammation and increase antioxidant defenses in elderly patients with ischemic stroke, compared to casein-containing formula [35]. Montaner et al. in a pilot, double-blind, randomized, multicenter clinical trial to study the efficacy of Simvastatin in the acute phase of ischemic stroke, evaluated the evolution of several inflammation markers [IL-6, IL-8, IL-10, monocyte chemoattractant protein-1, intercellular adhesion molecule-1, vascular cell adhesion molecule-1, C-reactive protein, sApo/Fas, tumor necrosis factor-alpha, E-selectin, L-selectin and nitrites + nitrates] and neurological outcome at baseline, day 1, 3, 5, 7 and 90. They found no differences among the biomarkers studied regarding treatment allocation. But the outcome of their patients was improved [36]. Raju et al. performed a pilot randomized controlled trial comparing the effect of colchicines (1 mg per day) with placebo on high sensitivity C-reactive protein (hsCRP) levels and platelet function in 80 patients with acute coronary syndrome or acute ischemic stroke who were followed for 30 days. Their study provided no evidence that colchicine suppresses inflammation in patients with acute coronary syndrome or acute ischemic stroke [37].

IMOD™, which is a combination of plant extracts enriched with selenium and has exhibited anti-inflammatory and immunoregulatory properties, has been studied in several trials evaluating its effects on different clinical situations. In a study by Mahmoodpoor et al., the effect of IMOD™ on controlling signs and symptoms and treatment of patients with sepsis was evaluated and it was reported that TNF-α levels, as a chief inflammatory mediator involved in sepsis, significantly decreased after treatment with IMOD™ compared to the control group [11]. Another study by Eslami et al. showed that Septimeb (IMOD™) had positive effects on survival of patients with severe sepsis. Considering withdrawal of Activated Protein C from market, IMOD™ might be considered as an adjuvant therapy for standard treatment of sepsis [38]. In addition, Baghaee et al. evaluated the effect of IMOD™ on inflammatory bowel disease in rats and reported that the medication significantly decreased TNF-α and IL-1β levels, resulting in a decrease in macroscopic tissue damage [12]. Also, the potential effects of IMOD™ have been shown in initial evaluations in HIV patients on decreasing TNF-α levels and decreasing TNF-α and IL-2 levels during patenting processes [39]. These studies showed that IMOD has anti-inflammatory effects.

In this context, IMOD™ as an immunomodulatory medication may be a therapeutic option in AIS. Vafaee et al. studied the neuroprotective effect of Setarud (IMOD™) on cerebral ischemia in male rats. In this randomized controlled animal study, rats intraperitoneally administered with Setarud after Middle Cerebral Artery occlusion. The study showed that Setarud could reduce infarct volume of subjected rats. The medication could alleviate degenerative changes in cortical neurons and also improved motor function of rats with cerebral ischemia [40].

In the present randomized clinical trial designed to assess the effect of IMOD™ on main inflammatory biomarkers, as reflexives of neuroinflammation in AIS, 99 patients with their first ischemic stroke attack were divided into two groups of control and IMOD™ and evaluated in relation to the levels of inflammatory markers in the first days of symptom initiation. The results showed that IMOD™ had a significant effect on some inflammatory markers, such as IL-6 and hsCRP, which was consistent with the clinical situation of patients in the case of hsCRP. In addition, the medication decreased TNF-α and IL-1 levels, although with no statistical significance. Previous studies have shown that IMOD™ can significantly reduce TNF-α and IL-1 levels [11,12,39],but insignificant effect of IMOD™ on these biomarkers in present study may be due to different amount or source of secretion of various biomarkers in AIS; although, further studies measuring biomarkers in serum and CSF may be needed to test this hypothesis. This study shows that IMOD™ has immunomodulatory effects as shown in previous studies.

Several studies have shown that C-reactive protein (CRP), an inflammatory marker, is associated with stroke severity and outcome [41]. Dewan et al., demonstrated that high CRP level is associated with stroke severity at admission and is an independent predictor of early seven-day mortality after ischemic stroke [42]. Hasan et al. showed three biomarkers (C-reactive protein, P-selectin and homocysteine) significantly differentiated between ischemic stroke and healthy control subjects [43]. Similarly, in a study by Idicula et al., a significant relationship was reported between a high CRP Level, a high NIHSS score and high mortality rate [44]. Rajeshwar et al. showed that hsCRP and NO levels predict the incidence of ischemic stroke and hsCRP is an independent prognostic factor of poor outcome at 3 months [45]. In the present study, there was a significant relationship between disease severity (NIHSS)at referral and an increase in hsCRP level on the first and the 4th days in both groups.

As in previous studies, we observed no major side effect of IMOD injection except for few cases of superficial phlebitis that resolved by conservative measures. In a study by Mahmoodpoor et al., it was shown that IMOD™ had no side effects on coagulatory factors such as platelets, prothrombin time, partial thromboplastin time, fibrinogen and D-dimer [10]. In our study changes in WBC and platelet counts subsequent to receiving IMOD™ were not significant, so, present study supports the safety of the drug.

### Limitation of the study

First, the number of included patients was small (limitation of eligible patients in that period of time). Second, a potential random error can’t be eliminated completely and our results remained to be confirmed. Third, placebo was not used in present study because of technical and ethical limitations.

## Conclusion

Considering the role of inflammation in the induction of ischemic cerebral damage, it appears that treatment with IMOD™ improves the profiles of main inflammatory markers; therefore, it might be considered as a therapeutic option in ischemic strokes. However, it is necessary to match laboratory findings with clinical findings and follow them in the long term to further elucidate such an effect. We recommend future multicenter studies with larger sample size and long duration period. Evaluation of the effect of the drug on the inflammatory profile in the cerebrospinal fluid (CSF) of experimental models may provide more localizing data about the effect of IMOD™.

## Competing interests

The authors reported no competing interest.

## Authors’ contributions

MF: Manuscript preparing, approved the final manuscript. MN-N: Study conduct, Manuscript preparation, approved the final manuscript. AM: Literature Review, Manuscript preparation, approved the final manuscript. MH: study conduct, approved the final manuscript. ES: study conduct, approved the final manuscript. BB: Analysis of biomarkers, approved the final manuscript. AT: Data analysis, approved the final manuscript, DS-O: Data analysis, approved the final manuscript. HS-B: Study conduct, approved the final manuscript. ES-h: Study conduct, approved the final manuscript. HA: Study conduct, approved the final manuscript. RR: Study conduct, approved the final manuscript. All authors read and approved the final manuscript.
